# Maxillary Bone Fracture Due to a Miniscrew-Assisted Rapid Maxillary Expansion: A Case Report

**DOI:** 10.3390/jcm14061928

**Published:** 2025-03-13

**Authors:** Ushio Hanai, Hiroyuki Muramatsu, Tadashi Akamatsu

**Affiliations:** 1 Department of Plastic and Reconstructive Surgery, Tokai University School of Medicine, Isehara 259-1143, Kanagawa, Japan; at7071@tokai.ac.jp; 2 Ichikawa Orthodontic Office, Shinwa-Kai Medical Corporation, 4-6-1-3F Koyasu-cho, Hachioji 192-0904, Tokyo, Japan; hero@shinwa.or.jp

**Keywords:** presurgical orthodontics, miniscrew-assisted rapid palatal expansion, complications, infraorbital nerve distribution, maxillary fractures

## Abstract

**Background/Objectives**: Miniscrew-assisted rapid palatal expansion (MARPE) has been increasingly used as a nonsurgical alternative for maxillary expansion in adults. However, reports of complications remain limited. This case describes a rare instance of maxillary bone fracture following MARPE and its clinical implications. **Methods**: A 32-year-old patient underwent MARPE as part of presurgical orthodontic treatment for maxillary constriction. Five days after activation, severe pain developed, followed by sensory disturbances in the infraorbital region. CT imaging revealed a maxillary fracture extending from the infraorbital foramen to the alveolar process. Symptoms gradually improved over two years, but psychological distress led to the abandonment of orthognathic surgery. **Results**: This case suggests that MARPE-induced maxillary fractures may be associated with stress concentration at the zygomatico-maxillary suture, particularly in individuals with increased midpalatal suture interdigitation and thin cortical bone. Finite element analysis and stress distribution studies indicate that the zygomatic buttress serves as a major resistance point, which may have contributed to the fracture. **Conclusions**: These findings highlight the importance of careful patient selection, preoperative CT assessments of bone thickness, and individualized expansion protocols. In high-risk cases, alternative approaches, such as surgically assisted expansion, may be considered. Further research on MARPE’s risk assessment and treatment protocols is needed to improve safety.

## 1. Introduction

Rapid palatal expansion (RPE) is a treatment method employed in orthodontic care for growing children when the maxillary dental arch needs to be expanded [1]. When forces larger than those used for ordinary tooth movement are applied to expand the maxillary arch, the midpalatal suture of the maxilla separates and stabilizes over time through localized bone growth and proliferation, resulting in the expansion of the maxillary alveolar arch width [2]. However, in adults, RPE alone is generally considered unsuitable due to the fusion of the midpalatal suture.

Instead, surgical-assisted rapid palatal expansion (SARPE), which combines orthodontic devices with surgical procedures, has traditionally been performed to achieve maxillary arch expansion in adults [3,4].

In recent years, miniscrew-assisted rapid palatal expansion (MARPE) has gained popularity, allowing for maxillary arch expansion in adults in the absence of surgical assistance [5]. MARPE has emerged as a nonsurgical approach to the treatment of transverse maxillary deficiency in late adolescents and adults. Unlike conventional RPE, which relies on tooth-borne anchorage and is the most effective in growing patients, MARPE utilizes skeletal anchorage through miniscrews inserted into the hard palate. This device utilizes screws implanted in the hard palate to facilitate maxillary alveolar arch expansion via midpalatal suture separation in adults. By applying forces directly to the maxillary bone, MARPE facilitates midpalatal suture separation and maxillary alveolar arch expansion, thereby achieving greater skeletal expansion while reducing undesirable dentoalveolar side effects such as buccal tipping, root resorption, and gingival recession [6,7]. Due to these advantages, MARPE has been widely adopted as an alternative to SARPE, offering a less invasive treatment option with fewer risks and lower patient morbidity [7,8]. Despite its increasing use, the safety and long-term effects of MARPE remain under investigation. The procedure is generally considered safe, with high success rates and minimal complications reported in the literature. Most documented issues involve minor side effects such as soft tissue irritation, temporary discomfort, or miniscrew loosening [8]. However, given the forces exerted on the midpalatal suture and surrounding structures, the potential for more severe complications, such as maxillary bone fractures, cannot be overlooked [6,9]. While cases of skeletal resistance leading to treatment failure have been described, reports of significant structural damage remain scarce. As MARPE continues to be used in a broader range of patients, including adults with increased midpalatal suture interdigitation, understanding its potential risks is critical for improving patient selection and management.

Herein, we report on a case of maxillary bone fracture caused by MARPE and discuss its implications. Although MARPE is widely regarded as a minimally invasive and safe alternative to surgical expansion, this case underscores the need for an increased awareness of its potential risks. Through this case report, we aim to highlight the necessity for further investigation into MARPE’s safety profile and provide insights for optimizing patient selection and treatment protocols.

## 2. Case Report

**Figure 1:**
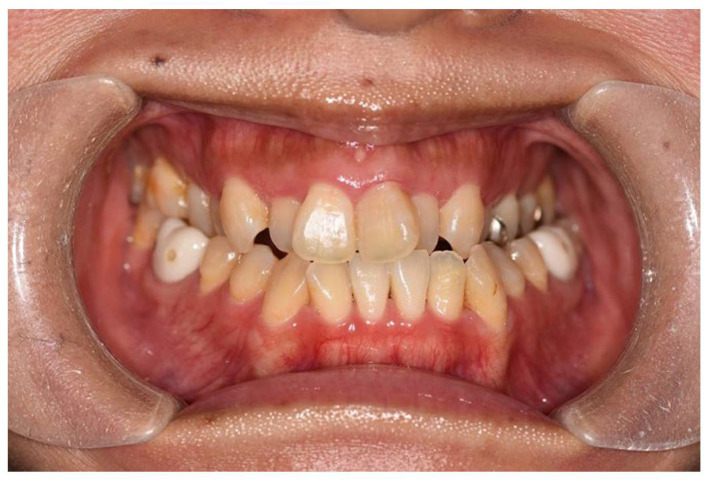
Initial occlusion at the first visit.

The patient was a 32-year-old female gym instructor who was referred to our plastic surgery department by an orthodontist for orthognathic surgery. She presented with Angle Class III malocclusion, an edge-to-edge bite with crowding, and maxillary arch constriction (Figure 1). An antero-posterior cephalometric analysis revealed an alveolar width discrepancy. In the study model analysis and coronal CT analysis, the distances between the functional cuspids were 37.9 mm and 47.9 mm in the maxilla and mandible, respectively. It was predicted that maxillary alveolar arch expansion through orthodontic treatment would be necessary to address the transverse arch discrepancy that would persist even after orthognathic surgery. A treatment plan was established to perform orthognathic surgery in our facility after leveraging presurgical orthodontic treatment to optimize the transverse dimension.

Angle Class III malocclusion, an edge-to-edge bite with crowding, and maxillary arch constriction were observed in the patient.

The appliance was fabricated with orthodontic bands on the first premolars and first molars. It features four holes for orthodontic screws, which were 11 mm long and inserted into the bicortical plates of the maxillary bone near the midpalatal suture.

**Figure 2:**
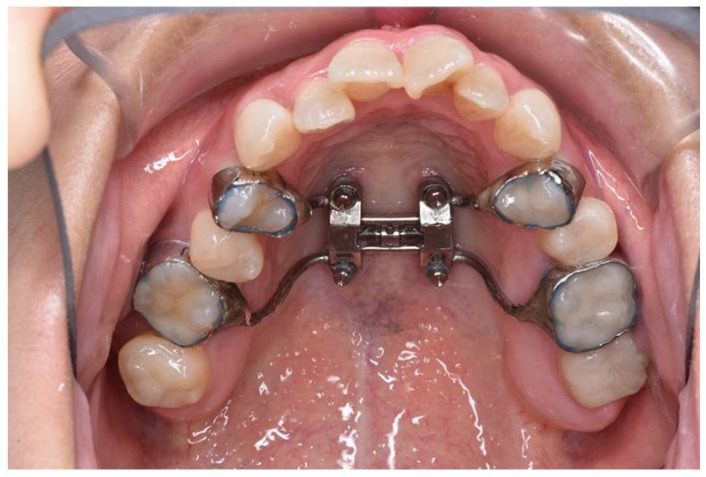
Intraoral view of patient with miniscrew-assisted rapid palatal expansion (MARPE) appliance in place.

The MARPE device (M.S.E., Maxillary Skeletal Expander, Forest-One Co., cChiba, Japan) was placed by the orthodontist. It was fabricated with orthodontic bands on the first premolars and first molars and designed with four holes for orthodontic screws. The expansion appliance was secured using 11 mm long screws inserted into the bicortical plates of the maxillary bone near the midpalatal suture (Figure 2). Subsequently, orthodontic treatment was initiated, and the patient also began maxillary expansion.

Comprehensive blood tests, including biochemistry, complete blood count, coagulation tests, infection screening, and imaging (electrocardiogram, chest X-ray), revealed no abnormalities. There were no findings suggestive of musculoskeletal metabolic disorders.

The MARPE device was activated as follows: 0.4 mm of expansion was performed daily, divided into two sessions, until a clear separation of the midpalatal suture was observed. After achieving suture separation, the expansion rate was reduced to 0.2 mm per day in a single session, aiming for a total expansion of approximately 7 mm.

Five days after the start of expansion, the patient suddenly experienced pain, and upon looking in the mirror, she noticed the widening of the midline of her dental arch. From that day, she felt severe pain requiring analgesics. On the morning of the 12th day, the patient experienced tingling sensory disturbances on the palatal side of the upper left anterior teeth, which extended to the nose and cheek later that day. By the evening of the same day, the sensation in these areas, as well as within her oral cavity, had completely disappeared. No facial swelling was observed at this time.

The patient’s orthodontist recommended discontinuing the expansion therapy.

The patient visited our plastic surgery outpatient clinic on the 20th day after starting expansion (15 days after symptom onset). Physical examination revealed that the sensation in her left upper lip had almost completely resolved. The sensation on the left side of the nose was entirely absent, while the sensation in the left cheek had slightly recovered but remained dull. The area inside the nasolabial fold and the left lower eyelid were completely anesthetic. No facial erythema or edema was observed. Based on these findings, we strongly suspected damage to the second branch of the left trigeminal nerve.

A separation of approximately 4 mm was observed in the midpalatal suture.

**Figure 3:**
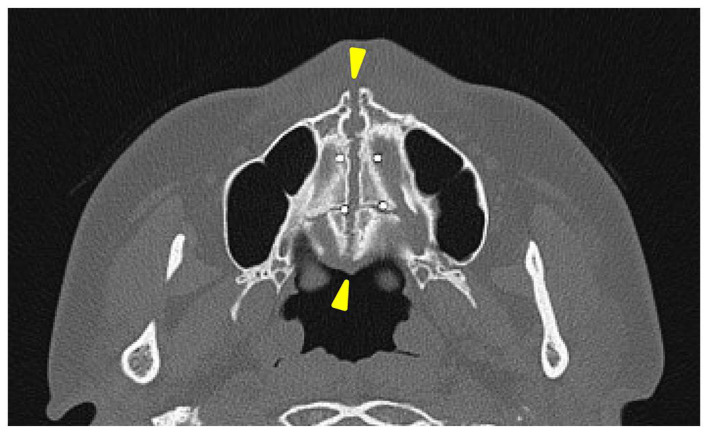
The axial view of the facial bone computed tomography at the level of the palatine bone. A separation of approximately 4 mm was observed in the area between the two yellow triangles in the midpalatal suture.

**Figure 4:**
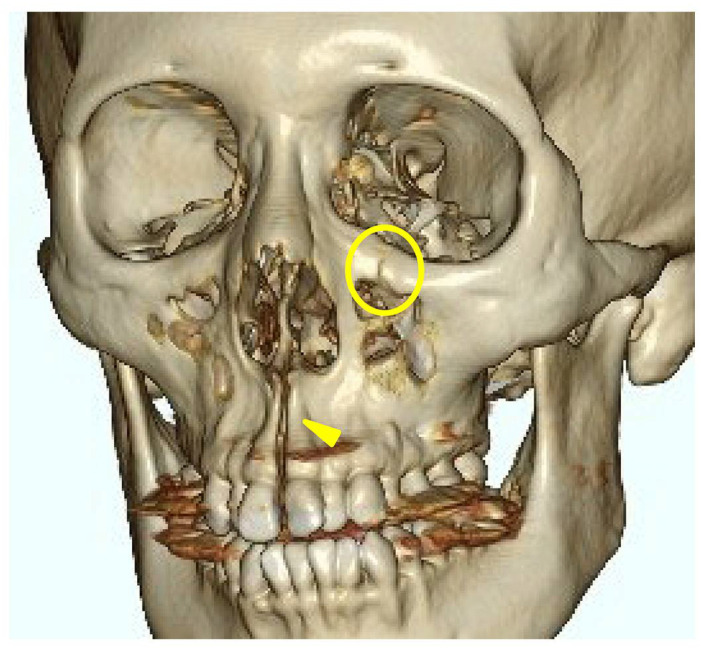
Three-dimensional computed tomography image of facial bones. A fracture line extending from the infraorbital rim to the infraorbital foramen (Circle mark) and a separation of the midpalatal suture (yellow triangle marks) were observed.

**Figure 5:**
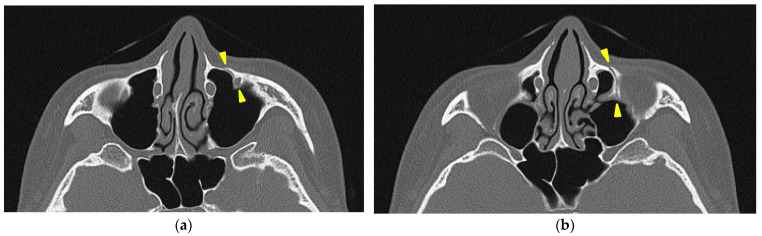
The axial view of the facial bone computed tomography at the level of the infraorbital canal. (**a**) A fracture line passing through the infraorbital canal is observed between the two yellow triangle markers. (**b**) A fracture line along the infraorbital canal is observed extending posteriorly to the orbital floor (yellow triangle marks).

A head CT was performed on the same day. The axial slices revealed a 4 mm wide separation of the midpalatal suture (Figure 3). A fracture of the left maxilla was also observed. The line of this fracture extended from the orbital floor and the anterior surface of the maxilla (Figure 4), starting at the medial edge of the orbital rim, passing through the entire wall of the infraorbital foramen (Figure 5a,b), and reaching the maxilla’s alveolar process.

The patient returned for a follow-up visit one month later, reporting improvement in symptoms despite the persistence of some sensory disturbances. At her two-month follow-up visit, there were no areas of complete anesthesia, although she continued to experience paresthesia in previously affected regions.

The sensory disturbances gradually resolved, and two years later, all symptoms had completely disappeared. However, the patient developed a phobia of the planned orthognathic surgery and ultimately canceled it, opting for orthodontic treatment alone.

## 3. Discussion

Reports of MARPE-induced maxillary bone fractures are unprecedented. However, fractures have been reported with SARPE. Shimming et al. [10] described the case of an alveolar process fracture in a 38-year-old woman undergoing SARPE due to midpalatal suture ossification. Thus, factors such as age and suture ossification can increase the risk of fractures, which may be even greater in nonsurgical expansions like MARPE.

The trigeminal nerve sensory disturbances observed in this case were likely caused by a fracture within the infraorbital canal and infraorbital foramen, resulting in nerve injury. This is supported by the fact that the symptoms were limited to the region innervated by the infraorbital nerve. The preservation of maxillary molar sensation indicates that the middle superior alveolar branch, which branches off before the infraorbital canal, was not damaged.

In this case, the fracture was concentrated around the zygomatico-maxillary suture for reasons that are unclear; however, there are studies investigating the correlation between the ossification state of facial bone sutures and the effects of rapid maxillary expansion (RME) devices. Provatidis et al. [11] analyzed the effects of RME on the craniofacial complex using finite element modeling, a computer simulation method, by applying RME to a dry human skull. Their analysis revealed that while the lacrimo-maxillary, fronto-maxillary, and naso-maxillary sutures had little influence on RME outcomes, the zygomatico-maxillary suture was significantly affected. In this case, three-dimensional CT reconstruction confirmed that the maxillary expansion was resisted by the surrounding bones, particularly the zygomatic bones, leading to the observed fracture. This finding suggests that, unlike in SARPE, where osteotomies reduce skeletal resistance, MARPE encounters direct resistance from surrounding craniofacial structures. Consequently, stress is concentrated at certain anatomical sites, potentially increasing fracture risk. This observation aligns with the finite element analysis by Provatidis et al., which demonstrated that the zygomatic bones serve as the primary limitation to maxillary expansion rather than the nasal or frontal bones. The presence of a fracture in the infraorbital region further supports this idea, as trauma-induced zygomatic fractures frequently involve fracture lines passing through the infraorbital foramen, leading to sensory disturbances similar to those observed in this case. Previous studies on trauma-induced zygomatic fractures have reported that fracture lines frequently pass through the infraorbital foramen, leading to sensory disturbances of the second branch of the trigeminal nerve, which are typical symptoms of such fractures [12,13]. The presence of a fracture in the infraorbital region in this case suggests that a similar mechanism may have contributed to MARPE-induced fractures, as the infraorbital foramen is inherently a site prone to fractures caused by external forces.

In this case, the expansion rate did not exceed the manufacturer’s recommendations. The maxillary bone fracture is likely caused by factors predisposing patients to fractures, such as stress concentration due to bone morphology, aging-related bone elasticity reduction, and suture ossification.

Additionally, recent studies on MARPE biomechanics have demonstrated that the height of the extender arms significantly affects the magnitude of force applied to the bone [14]. In particular, lower extender arms generate greater force loads, reaching up to 41.28 kgf (404.5 N) at the final activation. Compared to conventional tooth-borne expanders, which exert approximately 150 N, this force is significantly higher. This excessive force may exacerbate stress on critical resistance points such as the zygomatico-maxillary suture, especially in cases with thin palatal bone or advanced suture ossification. In such scenarios, stress overload may lead to microfractures or complete fractures, as seen in this case. The role of extender arm height in force distribution is therefore critical in treatment planning, and inappropriate extender arm selection may contribute to unexpected complications.

Furthermore, it has been reported that when cortical bone thickness is less than 0.62 mm, mini-implant retention strength decreases, increasing the risk of fracture under excessive loading [15]. This finding suggests that in patients with thinner palatal bone, the high force loads generated by MARPE—especially when using lower extender arms—may exceed the bone’s structural capacity, predisposing it to failure. Given that bone thickness varies significantly among individuals, a preoperative evaluation of cortical bone thickness should be considered essential in MARPE planning. In cases where cortical bone is found to be thin, alternative extender arm configurations or modified activation protocols may be necessary to reduce excessive stress and mitigate fracture risk.

The findings from SARPE studies also provide insight into the importance of force application and activation protocols in preventing complications. A systematic review [16] on SARPE has reported that slow activation protocols (≤0.5 mm/day) are associated with a higher incidence of asymmetric expansion, often necessitating surgical re-intervention. This is thought to be due to premature bone callus formation, which prevents uniform maxillary separation. While MARPE does not involve surgical osteotomies, it is possible that similar issues arise in cases with strong skeletal resistance, particularly in older patients with suture ossification. Consequently, the careful selection of the activation protocol is crucial to avoid asymmetric expansion and minimize stress concentration at vulnerable skeletal sites. Additionally, extender arm selection plays a vital role in optimizing force transmission, and inappropriate configurations may amplify existing resistance, leading to undesirable outcomes.

Currently, there is no established method for preoperatively assessing the risks of MARPE-induced fractures. However, the findings from stress analysis studies suggest that a preoperative CT evaluation of bone thickness and suture ossification is crucial for identifying high-risk cases. Considering the evidence from both SARPE and MARPE studies, it is clear that maxillary expansion success is dependent on both skeletal anatomy and biomechanical factors. In cases where preoperative imaging reveals extensive suture ossification or thin palatal bone, the careful selection of extender arm height and activation protocol is essential. If a high risk of fracture is identified, even SARPE may not be a viable option, and a surgical approach using multiple osteotomies during Le Fort I osteotomy should be considered. Therefore, for MARPE cases, a comprehensive evaluation of skeletal resistance, along with an individualized approach to device selection and activation protocol, is necessary to minimize complications and improve treatment outcomes.

## 4. Conclusions

This case report highlights a rare but clinically significant complication of MARPE, resulting in a maxillary bone fracture. The fracture, localized around the zygomatico-maxillary suture, appears to be associated with stress concentration during expansion. Finite element analysis and stress distribution studies suggest that the zygomatic buttress serves as a primary resistance point in maxillary expansion, which may contribute to localized stress overload in certain patients. Additionally, reduced bone elasticity and advanced suture ossification in adults could further increase fracture risk.

Given these factors, careful patient selection, including a preoperative CT evaluation of bone thickness and suture morphology, may help identify individuals at higher risk. The configuration of the MARPE device, particularly the height of the extender arms, should be tailored to the patient’s skeletal characteristics to optimize force distribution. In high-risk cases, presurgical orthodontic expansion should be reconsidered, and alternative approaches, such as surgically assisted expansion, may be more appropriate. In high-risk cases, presurgical orthodontic treatment must be avoided, and a surgical approach to transverse expansion should be considered. The clear communication of potential risks to the patient and close observation throughout treatment are crucial for reducing complications and improving patient outcomes. Further research into MARPE biomechanics, force application patterns, and patient selection criteria is necessary to enhance safety and effectiveness in clinical practice.

## Data Availability

The deidentified patient data supporting this study are available from the corresponding author upon reasonable request.

## Abbreviations

The following abbreviations are used in this manuscript:

MARPE	Miniscrew-assisted rapid palatal expansion
RME	Rapid maxillary expansion
RPE	Rapid palatal expansion
SARPE	Surgical-assisted rapid palatal expansion
